# Impact of the Phosphatidylinositide 3-Kinase Signaling Pathway on the Cardioprotection Induced by Intermittent Hypoxia

**DOI:** 10.1371/journal.pone.0076659

**Published:** 2013-10-04

**Authors:** Giuseppina Milano, Provvidenza Maria Abruzzo, Alessandra Bolotta, Marina Marini, Laura Terraneo, Barbara Ravara, Luisa Gorza, Maurizio Vitadello, Sabrina Burattini, Davide Curzi, Elisabetta Falcieri, Ludwig K. von Segesser, Michele Samaja

**Affiliations:** 1 Centre Hospitalier Universitaire Vaudois, Lausanne, Switzerland; 2 Laboratorio di Biologia Vascolare e Medicina Regenerativa, Centro Cardiologico Monzino, IRCSS, Milan, Italy; 3 Department of Experimental, Diagnostic and Specialty Medicine, University of Bologna, Bologna, Italy; 4 Department of Health Science, University of Milan, Milan, Italy; 5 Dipartimento di Scienze Biomediche, Università di Padova, Padova, Italy; 6 CNR Institute of Neuroscience, Padova section, Padova, Italy; 7 DiSTeVA, University of Urbino “Carlo Bo”, Urbino, Italy; University of Colorado Denver, United States of America

## Abstract

**Background:**

Exposure to intermittent hypoxia (IH) may enhance cardiac function and protects heart against ischemia-reperfusion (I/R) injury. To elucidate the underlying mechanisms, we developed a cardioprotective IH model that was characterized at hemodynamic, biochemical and molecular levels.

**Methods:**

Mice were exposed to 4 daily IH cycles (each composed of 2-min at 6-8% O_2_ followed by 3-min reoxygenation for 5 times) for 14 days, with normoxic mice as controls. Mice were then anesthetized and subdivided in various subgroups for analysis of contractility (pressure-volume loop), morphology, biochemistry or resistance to I/R (30-min occlusion of the left anterior descending coronary artery (LAD) followed by reperfusion and measurement of the area at risk and infarct size). In some mice, the phosphatidylinositide 3-kinase (PI3K) inhibitor wortmannin was administered (24 µg/kg ip) 15 min before LAD.

**Results:**

We found that IH did not induce myocardial hypertrophy; rather both contractility and cardiac function improved with greater number of capillaries per unit volume and greater expression of VEGF-R2, but not of VEGF. Besides increasing the phosphorylation of protein kinase B (Akt) and the endothelial isoform of NO synthase with respect to control, IH reduced the infarct size and post-LAD proteins carbonylation, index of oxidative damage. Administration of wortmannin reduced the level of Akt phosphorylation and worsened the infarct size.

**Conclusion:**

We conclude that the PI3K/Akt pathway is crucial for IH-induced cardioprotection and may represent a viable target to reduce myocardial I/R injury.

## Introduction

Ischemic preconditioning (IP), term used to identify the phenomenon whereby repeated exposures to sub-lethal ischemia episodes enhance myocardial protection against potentially lethal ischemia [1], probably still represents the best tool for efficient prevention strategy against cardiovascular diseases. IP is composed of an immediate (hours) and a delayed (days) phase [2]. Whereas the first is caused by rapid posttranslational modification of existing proteins, the latter, also called “second window of protection” or SWOP, provides sustained protection as a result of persisting changes in the expression of proteins involved in protection [3]. Although IP is a potentially powerful and economic approach to reduce the burden of myocardial ischemia disease, several bottlenecks prevent its usability in humans. Exercise training is nevertheless a recognized strategy to induce delayed IP [4]. However, recommending exercise training is not a feasible option for some patients, including individuals with physical disabilities or exercise-intolerant patients, who may therefore be considered at high risk for cardiovascular diseases. Alternative non-invasive techniques to induce IP are therefore envisaged.

A procedure that is gaining popularity among athletes because of promising results in terms of fitness and endurance gain, intermittent hypoxia (IH) is currently under scrutiny for prevention of cardiovascular diseases as well. IH was shown to increase exercise tolerance in elderly men with and without coronary artery disease [5] and to improve myocardial perfusion in coronary patients [6]. However the underlying mechanisms, which may be responsible for the marked dichotomy in the effects of IH on the cardiovascular system, remain elusive: besides representing a cardioprotective paradigm, IH is also associated to overt myocardial damage in patients suffering from obstructive sleep apnea (OSA). The protective vs. damaging effects of IH were examined elsewhere [7]: possibly the three-phase time-dependent effect modulated by GATA-4-dependent gene transcriptional regulation via inhibition of histone deacetylase play an important role [8]. But the characterization of the transformation of IH from a cardioprotective into an adverse factor is beyond the purposes of the present study, which mainly focuses into the cardioprotective effects of IH.

Protein kinase B, or Akt, plays an important role in the phosphatidylinositide 3-kinase (PI3K) signaling pathway. When activated through translocation to the membrane and phosphorylation on either Thr-308 or Ser-473, Akt stimulates a variety of targets and acts as a nodal regulatory kinase in myocardium [9], mainly with pro-survival, proliferative and protective effects. The purpose of this study is to develop a cardioprotective IH model to identify the underlying mechanisms at morphological, biochemical and molecular levels.

To address cardioprotection, we adopted a validated method [10]. Angiogenesis was addressed by measuring the vascular endothelial growth factors (VEGFs), a family of glycoproteins involved in the regulation of vasculogenesis. The hypoxia-inducible factor (HIF)-1α stimulates VEGF expression by direct transcriptional activation and through the PI3K/Akt signaling pathway [11]. VEGF binds to tyrosine kinase receptors (VEGF-Rs), one of which, VEGF-R2/flk-1, whose synthesis occurs through an autocrine mechanism [12], is known to mediate the majority of the cell responses to VEGF-A, the most important VEGF isoform. To address the effects of IH on the oxidative stress, we measured the expression of the inducible isoform of the major heat shock protein, HSP70, due to its known protective role during acute hypobaric hypoxia [13] and exercise training [14]. Wortmannin, a fungal metabolite that irreversibly binds and inhibits mammalian PI3K with an IC_50_ in the low nanomolar range, thereby preventing Akt phosphorylation [15], provides a unique way to test the involvement of the PI3K signaling pathway in this situation.

## Materials and Methods

Animal experiments were performed in accordance with the Swiss federal law and with the Guide for the Care and Use of Laboratory Animals published by the US National Institutes of Health (NIH Publication No. 85-23, revised 1996). The protocol was approved by the Committee of the Ethics of Animal Experiments of the Centre Hospitalier Universitaire Vaudoise (Permit Number 2262). All surgery was performed under ketamine-xylazine anesthesia and all efforts were made to minimize suffering.

Experiments were conducted on 8-10-wk old male C57Bl6 mice (Charles River, France). Mice were fed standard diet without limitations until 24 h before sacrifice. Room temperature was kept at 21±2°C and 12 h of light were alternated to 12 h of dark.

### Intermittent hypoxia protocol

**Figure 1:**
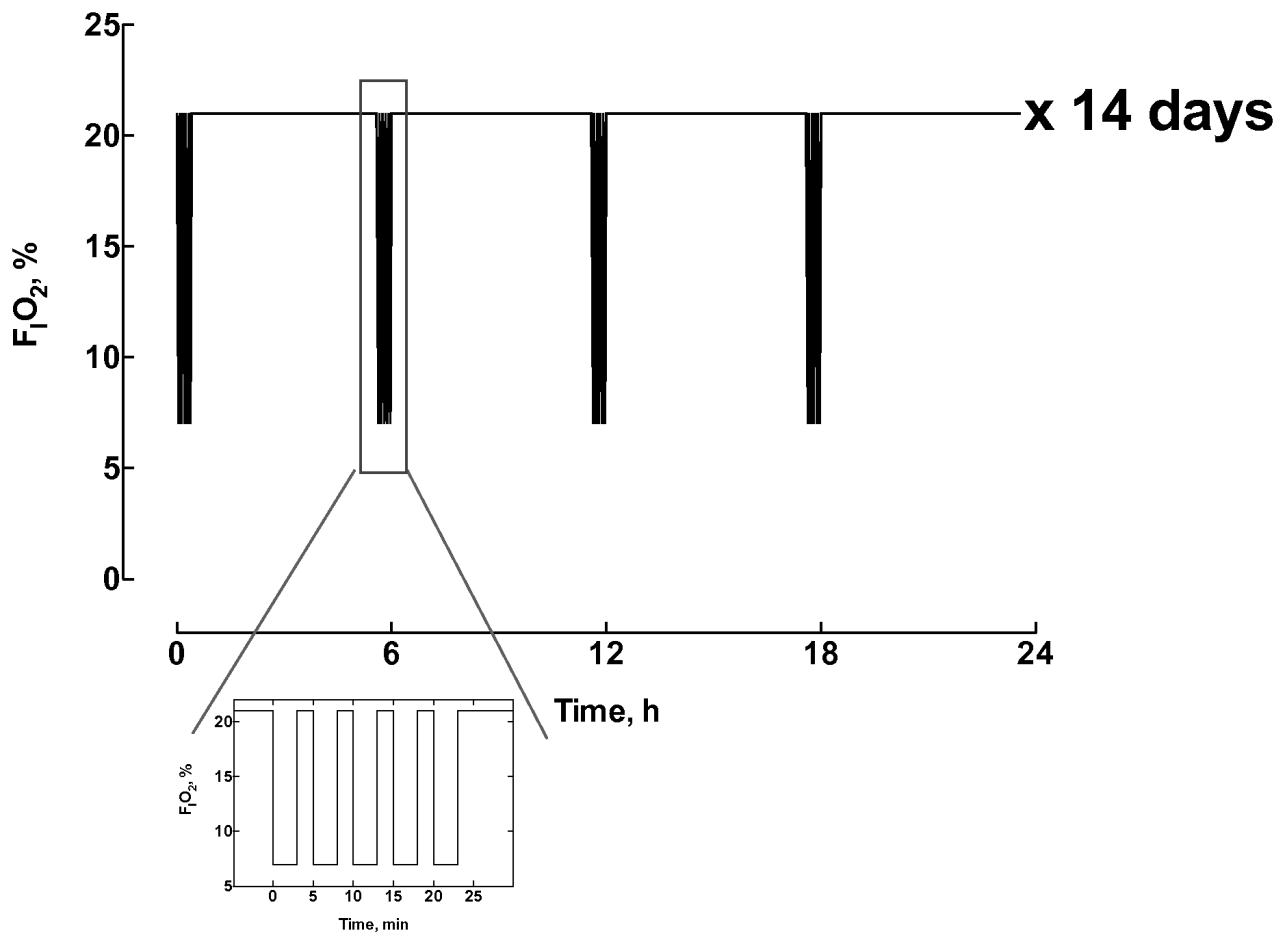
Scheme of the intermittent hypoxia protocol used in this study. FIO_2_: fraction of O_2_ in inspired air (%).

Mice were placed in gas-tight Plexiglas boxes (40x20x20 cm) equipped with a Clark-type O_2_ electrode and flushed with room air by means of a gas pump. A system of computer-assisted solenoid valves was operated to distribute air or N_2_ to the chamber. At the beginning of the IH events, the chambers were flushed with N_2_ for 3 min to achieve 6-8% O_2_ followed by 2 min air to restore 21% O_2_. Each cycle was repeated 5 times and each IH train was applied 4 times/day for 14 days (5 h 35 min between two successive IH trains, Figure **1**). A total of 32 mice were exposed to IH, while 40 were maintained in control conditions (air in the place of N_2_). Three hours after the last IH train, mice were anesthetized by intraperitoneal injection of ketamine (150 mg/kg) plus xylazine (5 mg/kg), and randomly assigned to a group of tests. In additional control or IH mice (10/10), the PI3K inhibitor wortmannin was administered intraperitoneal (24 µg/kg) 15 min before ischemia.

### Left ventricular Pressure-Volume (P-V) loops analyses

Hemodynamic parameters were collected in 7/6 control/IH mice. The anesthetized mouse was placed in a supine position on a heating pad at 37.5°C. The trachea was cannulated and the animal attached to a positive-pressure volume controlled rodent ventilator (MiniVent, Hugo Sachs Elektronik, Harvard Apparatus, March-Hugstetten, Germany). The tidal volume and the ventilatory rate were set at 200-250 µl and 110-130 breaths/min, respectively. The right carotid artery was dissected and a Millar Mikro-Tip conductance catheter (model SPR-839, tip size of 1.4F, Millar Instruments, Oxford, UK) introduced into the artery and advanced into the LV via the aortic valve [16]. Once steady-state hemodynamic was achieved, P-V loops were recorded and processed using an MPVS Ultra system (Millar Instruments, Oxford, UK). The cuvette calibration method (Millar Instruments) was used to calculate the absolute volume data.

### LAD ligature

Myocardial infarction was induced by regional left anterior descending (LAD) coronary artery occlusion in 15/12 control/IH mice (5/5 of these were administered with wortmannin). After anesthesia, the trachea was cannulated and mice ventilated as described above. A left thoracotomy was performed between the 4th and 5th rib and the heart was exposed to pass a 7-0 silk at 2-mm below the tip of the left auricle. The suture was tied using a shoestring knot over a 1-mm polyethylene tube (PE-10). After 30 min ischemia [17], the LAD was reopened for 3 h by pulling on the exteriorized suture to release the knot and the heart reperfused. Of all hearts subjected to this procedure, 8/5 control/IH hearts were used for the measurement of proteins carbonyl groups (see below), whereas 10/7 hearts were used to measure the area at risk and the infarct size.

### Area at risk and infarct size

At the end of the reperfusion, the LAD was re-occluded and 5% Evans blue (250 µl) was injected into the left ventricle to mark the ischemic zone as tissue area without the blue dye. The heart was frozen in liquid N_2_ and stored at -20°C until analysis. To measure the infarct and risk areas, the heart was cut into five/six 1-mm thick transverse slices from apex to base. The slices were incubated in 1% triphenyltetrazolium chloride in sodium phosphate buffer at 37°C for 20 min to stain viable cells in the risk zone. Afterwards, the slices were immersed in 10% formalin for 24 h to enhance contrast between stained and unstained areas, with the latter representing the infarct size. The extent of stained and unstained areas was calculated for each slice from computerized images using NIH Image software. The risk area is expressed as percentage of total ventricle area whereas the infarct area is expressed as a percentage of the risk area.

### Myocardial morphology

For morphologic measurements in 4/4 control/IH freshly removed hearts, excess water was absorbed on paper, atria were excised and the ventricles plus the septum were weighed using a precision balance. In additional 5/4 control/IH mice, small fragments (<1 mm^3^) of the left ventricle tip were quickly fixed with 2.5% glutaraldehyde in 0.1 M phosphate buffer, post-fixed with 1% OsO4 in the same buffer, dehydrated with alcohol, and embedded in araldite. Semi-thin sections stained with 1% toluidine blue in distilled water at 60°C, were used to evaluate the number of capillaries/mm^2^. For each heart, seventy-five non-overlapping areas (800x magnification) were digitalized and analyzed making use of the Olympus BX51 microscope with the Olympus C-3030 digital camera and the Cell^B software. The total surface evaluated per condition was 1.44 mm^2^. The number of capillaries was evaluated following the visual identification of endothelial cells, as previously described [18]. Thin sections, stained with uranyl acetate and lead citrate, were observed with a transmission electron microscope (Philips CM10).

### Myocardial tissue analyses

In 6/6 control/IH mice, freshly removed hearts were quickly rinsed in ice-cold PBS (pH 7.4), clamped between steel tongs pre-cooled with liquid N_2_ and stored at -80°C until analysis. For Akt assays, we also evaluated 5/5 control/IH mice treated with wortmannin.

### Western blot

The expression level of target proteins was analyzed by Western blot technique. Frozen left ventricles were lysed in a buffer containing 10 mM TRIS-HCl, 50 mM KCl, 1 mM EDTA, 1% NONIDET P-40 and 3% Protease Inhibitor Cocktail (Sigma, St. Louis, MO, USA). Protein concentration was measured using either the Protein Assay or the Coomassie Blue kit (Bio-Rad Laboratories, Hercules, CA) according to manufacturer’s instructions. Equal amount of protein (40 µl) were separated on SDS-PAGE gel, electroblotted onto nitrocellulose membranes (Bio-Rad Laboratories, Hercules, CA). After blocking in Tris-Buffered Saline (TBS) containing 0.1% Tween-20 (TBS-T) and 5% of non-fat milk for 1 h at room temperature, membranes were probed overnight at 4°C with the following primary antibodies: rabbit polyclonal anti-VEGF-A (Calbiochem, Nottingham, UK); rabbit monoclonal anti-VEGF-R2 (Cell Signaling Technology, Danvers, MA); mouse monoclonal anti-α-actinin (Sigma, St. Louis, MO, USA); rabbit anti-α-tubulin (Santa Cruz Biothechnology); anti-Grp94 (clone 3C4 [19]); anti-Hsp70 (SPA-810, Stressgen); anti-HO-1 (OSA-110; Stressgen); anti-eNOS and P-eNOS (Ser^1177^) (Santa Cruz Biotechnology); anti-Akt and P-Akt Ser^473^ (Cell Signaling Technologies Abs); rabbit anti HIF-1α (Santa Cruz Biotechnology); and rabbit anti-Nrf2 (Santa Cruz Biotechnology). After washing three times with TBS-T, secondary antibodies were goat anti-mouse or goat anti-rabbit IgG (H+L) (Pierce, Rockford, IL), conjugated to horseradish peroxidase for 1 h at room temperature. Horseradish peroxidase reaction was detected using an enhanced chemiluminescent substrate, Super Signal West Dura Extended Duration (Pierce, Rockford, IL). Bands were scanned and their density quantified by means of BioRad GelDoc 2000 with reference to α-actinin or α- tubulin as loading controls.

### CHOP immunolocalization

Double immunofluorescence was performed to visualize translocation of the CHOP transcription factor into cardiomyocyte nuclei. After fixation and permeabilization as described [19], cryosections were incubated for 1 h at room temperature with a mixture of rabbit polyclonal anti-CHOP antibodies (R-20 or F-168, Santa Cruz Biotech) and mouse monoclonal anti-α sarcoglycan antibody (Monosan) to decorate cardiomyocyte sarcolemma. After adequate rinses, sections were incubated overnight at 4C with species-specific fluorescein and Texas Red conjugated secondary antibodies obtained in goat (Santa Cruz Biotech) and centrifuged before use to eliminate aggregates. Section were rinsed, mounted with buffered glycerol containing 4 µg/ml DAPI and observed using the Zeiss Microscope Axioplan equipped with epifluorescence optics. Images were acquired using a Leica digital DFC 300FX camera and the IM50 software (Leica Microsystems SRL, Milano, Italy). Three to four micrographic fields were collected consistently from sub-endocardial and sub-epicardial regions of each sample in order to evaluate at least 300 cardiomyocyte nuclei per section.

### Protein carbonyl groups

In 8/5 control/IH mice we measured the amount of protein carbonyl groups. This analysis was also performed in 8/5 control/IH mice after LAD ligature and reperfusion. Pre- and post-LAD hearts were frozen in liquid N_2_ and protein carbonylation analyzed using the OxyBlot protein oxidation detection kit (Millipore) as previously described [20]. In brief, about 10 sections (12 µm thickness) were cut transversally from the left ventricle wall. About 12 µg protein was used for derivatization with DNPH. The degree of protein carbonylation was determined after normalization with the amount of loaded proteins, evaluated by densitometry of the Ponceau Red staining.

### Statistics

Data are expressed as mean±SEM. Significance level was P=0.05 (two-tailed). To detect differences among the groups, we routinely perform one-way ANOVA followed by the Neuman-Keuls post-test. When two groups are compared, this test reduces to the Student’s t-test.

## Results

### Intermittent hypoxia improves myocardial contractility

**Figure 2:**
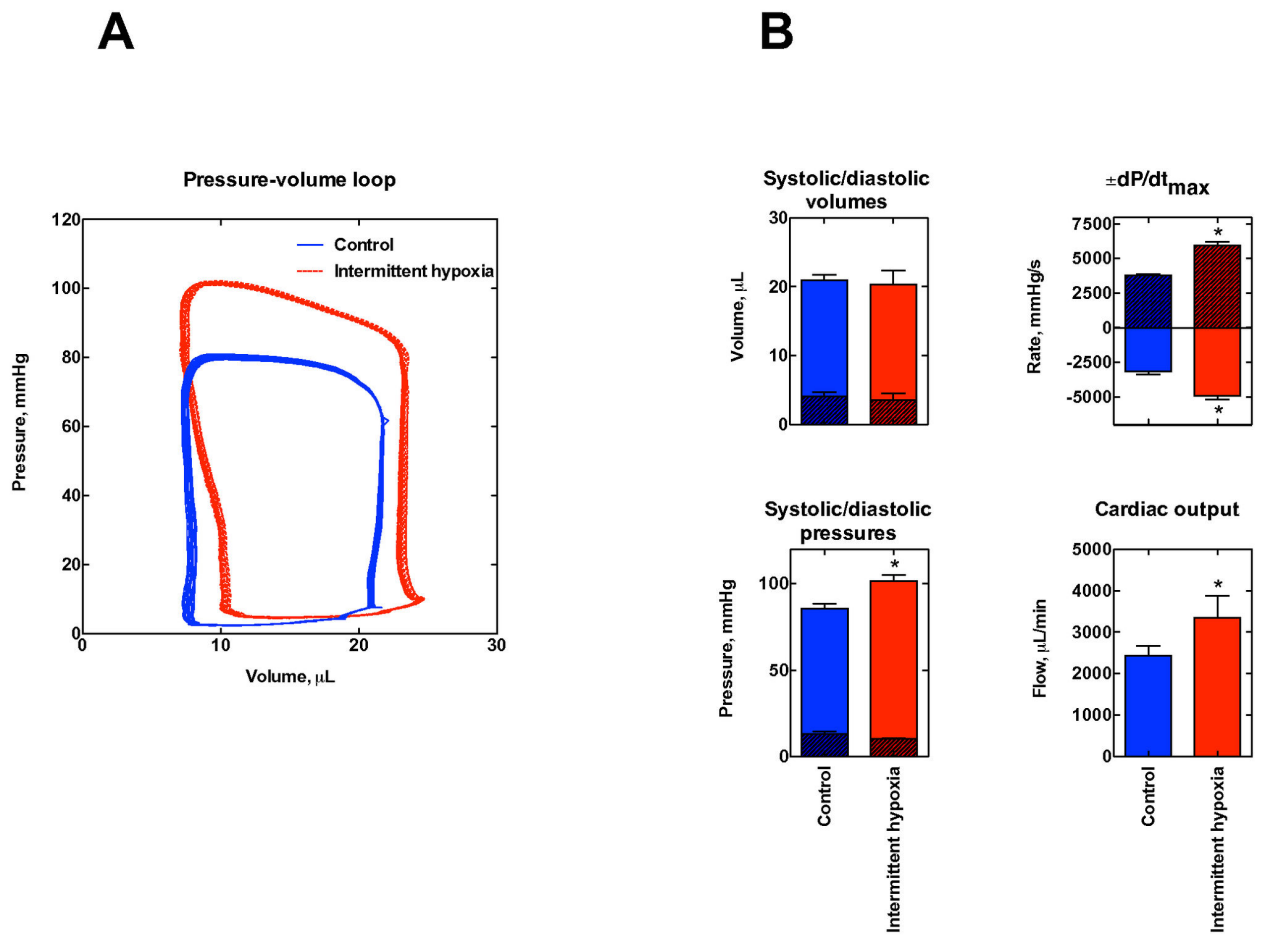
Hemodynamic data. Panel **A**. Pressure-volume loops obtained in ten consecutive contractions in two representative mice from either group. Panel **B**. Some hemodynamic data (mean±SEM) obtained in all the examined mice (n=7/6 control and intermittent hypoxia, respectively), * marks P<0.05 with respect to control, Student’s two-tailed t-test. The diastolic (clear) and systolic (shaded) volumes, systolic (clear) and diastolic (shaded) pressures, +dP/dt_max_ (shaded) and -dP/dt_max_ (clear), and the cardiac output (clear) are reported.

IH mice showed a modest albeit significant decrease in body weight compared to control (Table **1**). As heart weight also decreased in IH, the heart/body weight ratio was the same in the two groups, indicating lack of cardiac hypertrophy. Hematological parameters were maintained in all groups (not shown). Hemodynamic data and the pressure-volume loop curves measured in control and intermittent anesthetized mice (Figure **2**) show that exposure to IH did not affect neither the end-systolic nor the end-diastolic volumes. Likewise, the end-diastolic pressure was only marginally reduced by IH with a significant increase in end-systolic pressure respect to control. IH also induced a significant increase and decrease in maximum and minimum derivative pressures, respectively, compared to control. As a final result, the cardiac output increased from 2437, SE 233 to 3345, SE 528 µl/min (P=0.03), e.g. a 27% increase, indicative of markedly improved myocardial performance as an outcome of the IH treatment.

**Table 1:** Animal data and characteristics (mean±SE).

	**Control**			**Intermittent hypoxia**		
	Mean	SE	n	Mean	SE	n
*Body weight*						
initial, g	21.52	0.25	50	21.48	0.20	42
final, g	22.50	0.31	50	20.30	0.39*	42
change, g	0.98	0.21	50	-0.39	0.14*	42
*Heart*						
Heart weight, mg	98.1	4.1	7	87.0	1.2*	6
Heart/body weight, mg/g	4.50	0.12	7	4.23	0.08	6

*, p<0.05 (Student’s t-test).

### Intermittent hypoxia induces *neo-*angiogenesis through VEGF-R2 signaling

**Figure 3:**
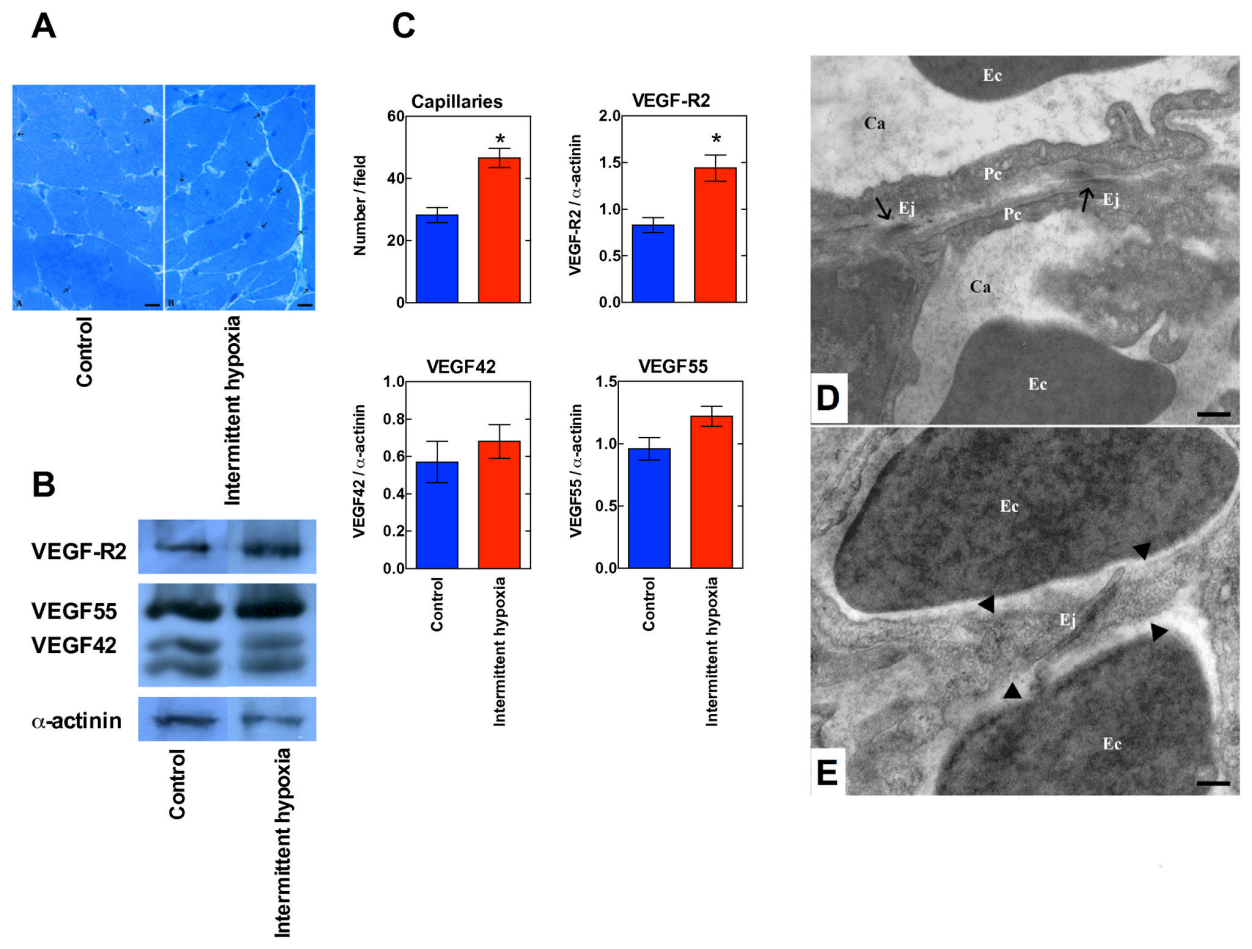
Angiogenesis. Panel A. Representative semithin sections stained with toluidine-blue from the left ventricle of a control and an IH mouse. The arrows indicate endothelial cells. The horizontal bar represents 10 µm. Panel B. Representative Western blots reporting the protein expression of VEGF-R2, VEGF55, VEGF42 as well as α-actinin (loading control). Panel C. Data related to angiogenesis as obtained in all available hearts (mean±SEM, n=6/6 control and intermittent hypoxia, respectively), * marks P<0.05 with respect to control, Student’s two-tailed t-test. The capillary count per unit area, as well as the expression of VEGF-R2, VEGF isoforms VEGF42 and VEGF55 as normalized for α-actinin (Western blot) are reported. Panels D and E. Transmission electron microscopy images of left ventricle sections from an IH mouse. In panel C, arrows (→) indicate endothelial junctions. In panel D, arrow heads (►) show a cellular “bridge” that partitions a pre-existing capillary in the process of neo-angiogenesis. Ca, capillary lumen; Ec, erythrocytes; Ej, endothelial junctional complexes; Pc, pericyte. The horizontal bar represents 0.25 µm.

Toluidine-blue staining shows a marked increase in the number of endothelial cells per unit area in IH with respect to control (Figure **3A**), indicative of neo-angiogenesis. The average capillary count per unit area increased markedly in left ventricles from IH mice with respect to control (P=0.03, Figure **3B-C**). Likewise, the expression of the vascular endothelial growth factor (VEGF) receptor 2 (VEGF-R2, also known as KDR/Flk-1) was increased in IH vs. control hearts (P=0.009). Whereas the protein expression of 42 kDa isoform of VEGF (VEGF42, i.e., the soluble fraction) was unaffected by IH, the 55 kDa isoform (VEGF55, i.e., the membrane-bound fraction) was slightly over-expressed, but without statistical significance (P=0.07, Figure **3B-C**). Transmission electron microscopy (Figure **3D-E**) further supports increased capillary count in IH hearts. The progressive appearance of a cellular “bridge” is visible: pericytes appear to be involved in the formation of an intercapillary pillar. Junctional complexes are formed between opposite pericyte walls. In panel E, pillar size is similar to that in D. The merging of the lateral walls has completed the “bridge” structure.

### Intermittent hypoxia slightly enhances the oxidative stress

**Figure 4:**
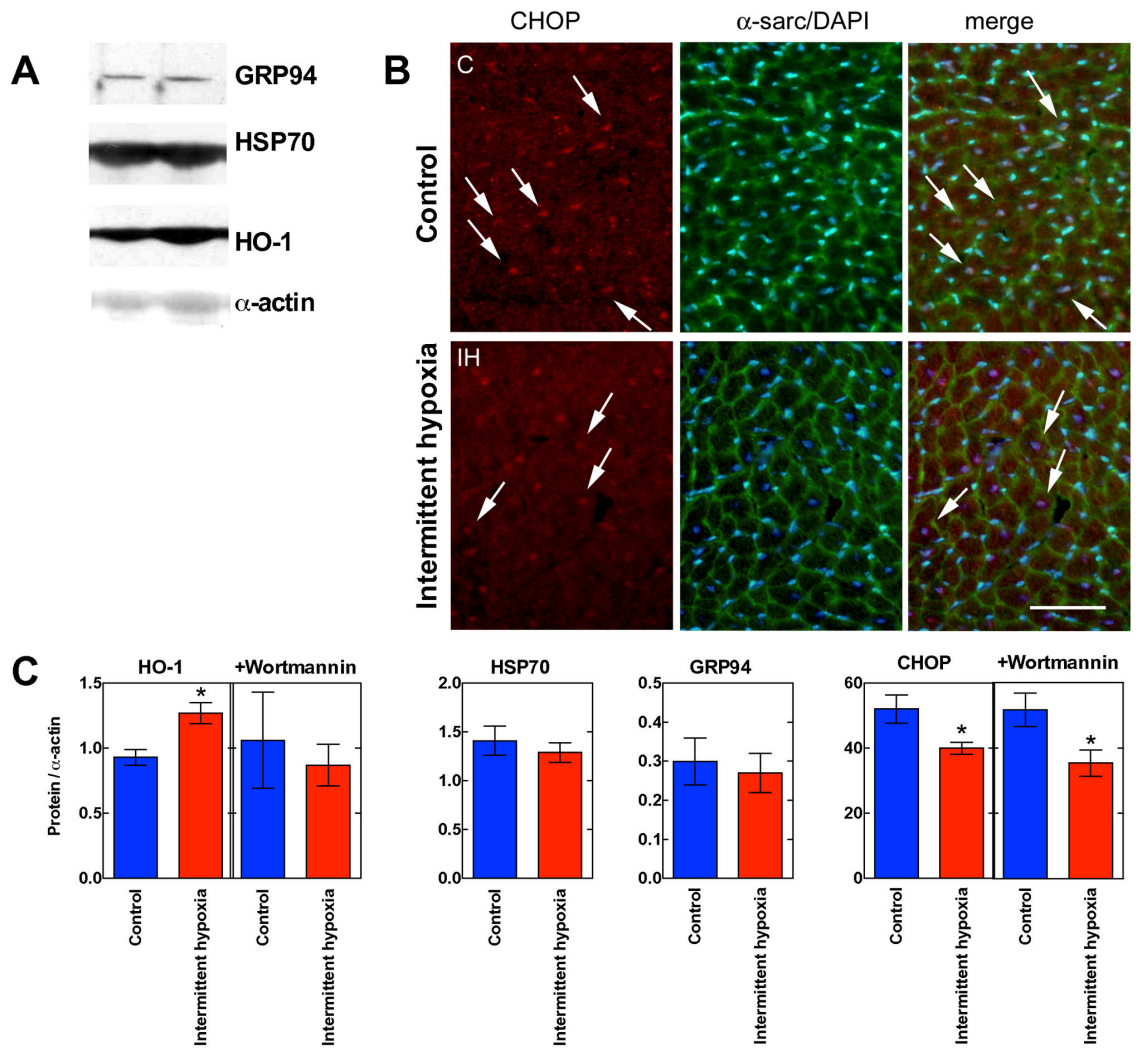
Stress proteins. Panel **A**. Sample Western blot images illustrating the protein expressions of HO-1, HSP70, GRP94 (referred to α-actin). Panel **B**. Double immunofluorescence analysis to investigate the nuclear localization of the transcription factor CHOP. The left column shows representative staining using anti-CHOP antibodies (red fluorescence), whereas the middle column shows α−sarcoglycan antibodies (green fluorescence). Nuclei were counterstained with DAPI (blue fluorescence). Arrows indicate cardiomyocyte nuclei positive for CHOP staining, which appears pink when merged with DAPI (right column). The upper and lower rows report a sample control and intermittent hypoxia heart. Bar: 50 µm. Panel **C**. Quantification of stress proteins from densitometry analysis of Western blots and analysis of Panel B images in all available samples (mean±SEM, n=6/6). *, P<0.05 with respect to control, Student’s two-tailed t-test. This panel also shows the effect of wortmannin on the expression of HO-1 and CHOP (n=5/5).

IH increased the protein expression of heme oxygenase-1 (HO-1, Figure **4A, C**). This increase was blunted in mice treated with wortmannin, in analogy with the effect led by this PI3K inhibitor on Akt phosphorylation (see later). By contrast, the expression level of Heat Shock Protein 70 kDa (HSP-70) and glucose-regulated protein 94 (GRP94), stress-proteins that were demonstrated to be up-regulated by hypoxia [21,22], remained unchanged. The pro-apoptotic factor endoplasmic reticulum stress-induced transcription factor C/EBP homologous protein (CHOP) was decreased by IH, without any effect led by wortmannin.

To assess whether HO-1 expression was increased consequently to an endoplasmic reticulum-stress response, a double immunofluorescence analysis was performed to investigate the nuclear localization of the transcription factor CHOP (Figure **4B**). In control hearts, about half of cardiomyocyte nuclei, identified by means of a nuclear fluorescent stain and circumscribed by α-sarcoglycan-positive sarcolemma, showed positive labeling for CHOP. This number appeared reduced in IH. To quantitatively assess this reduction, we considered 300 cardiomyocyte nuclei per heart in 5/5 control/IH hearts and measured the percentage of nuclei showing CHOP labeling (49.16±4.04%, and 38.00±2.00%, respectively, P=0.03). Thus cardiomyocyte nuclei with CHOP labeling were reduced by about one-third in IH. In mice treated with wortmannin, the percentage of nuclei showing CHOP labeling changed to 51.75±5.19%, and 35.33±4.07%, respectively (P=NS from non-treated mice), showing that the pattern was not affected by wortmannin (microphotographs not shown).

### Intermittent hypoxia potentiates PI3K-Akt signaling

**Figure 5:**
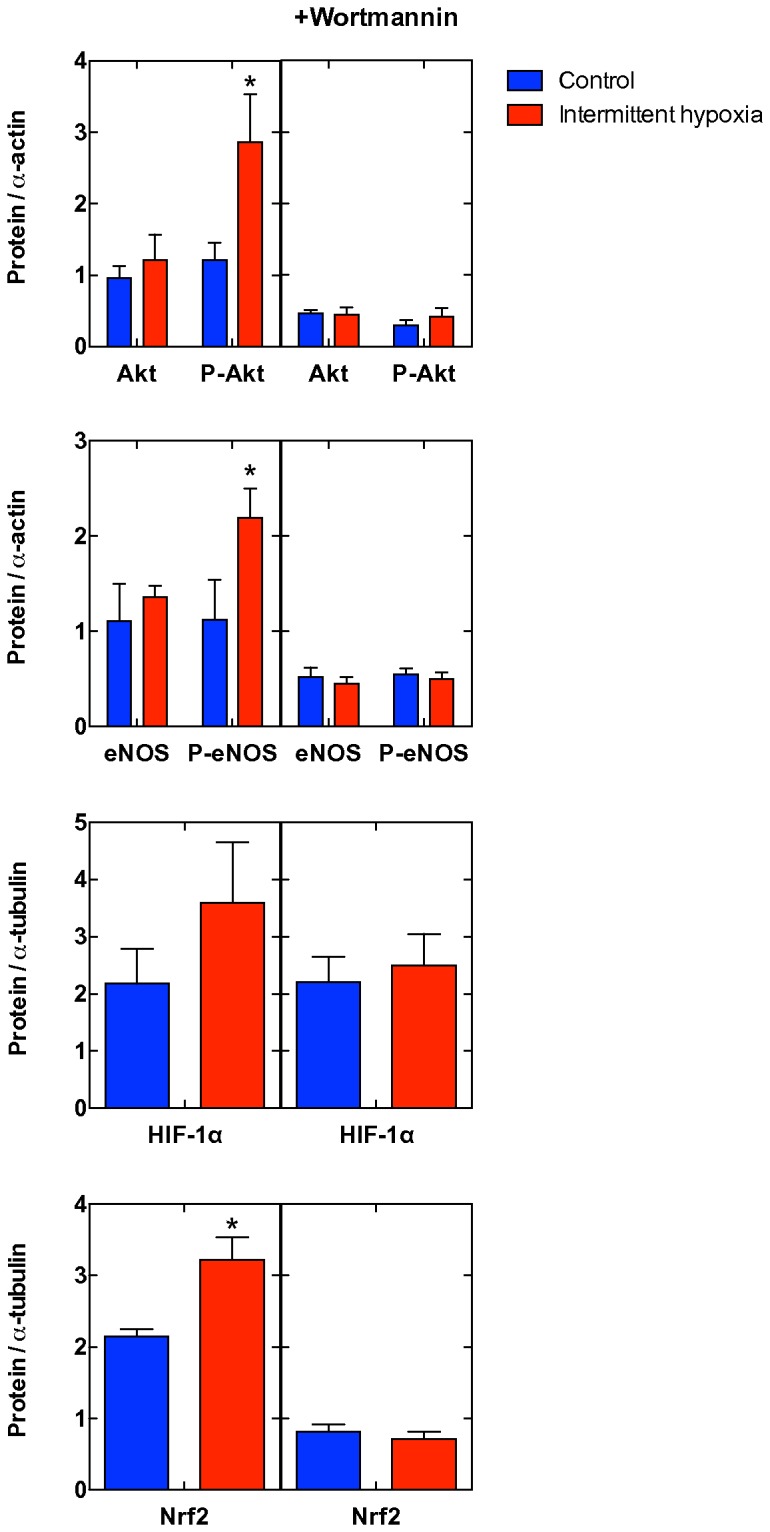
Signaling. Western blots of Akt, its phosphorylated isoform P-Akt, eNOS, its phosphorylated isoform P-eNOS, HIF-1α and Nrf2 in all available samples (mean±SEM, n=6/6 and 5/5 without and with wortmannin, respectively). *, P<0.05 with respect to control, Student’s two-tailed t-test.

Whereas the expression of Akt was not affected by IH, P-Akt expression was roughly 2.5 times higher in IH hearts (Figure **5**). The inhibitor wortmannin markedly reduced the expression of Akt and its phosphorylation in both control and IH hearts. The phosphorylation of the endothelial isoform of NO synthase (eNOS, or NOS3) is known to depend on the activity of Akt. In support of this, P-eNOS was markedly increased in IH but maintained in control hearts, whereas treatment with wortmannin blunted both eNOS expression and phosphorylation. The hypoxia-signaling path did not appear prominent and the expression level of HIF-1α was unchanged in control and IH hearts, and the treatment with wortmannin did not affect its expression. By contrast, IH increased the expression of the oxidative stress transducer nuclear factor (erythroid-derived 2)-like 2 (Nrf2), and the treatment with wortmannin greatly reduced Nrf2 expression both in control and IH hearts.

### Intermittent hypoxia-induced cardioprotection depends on Akt

**Figure 6:**
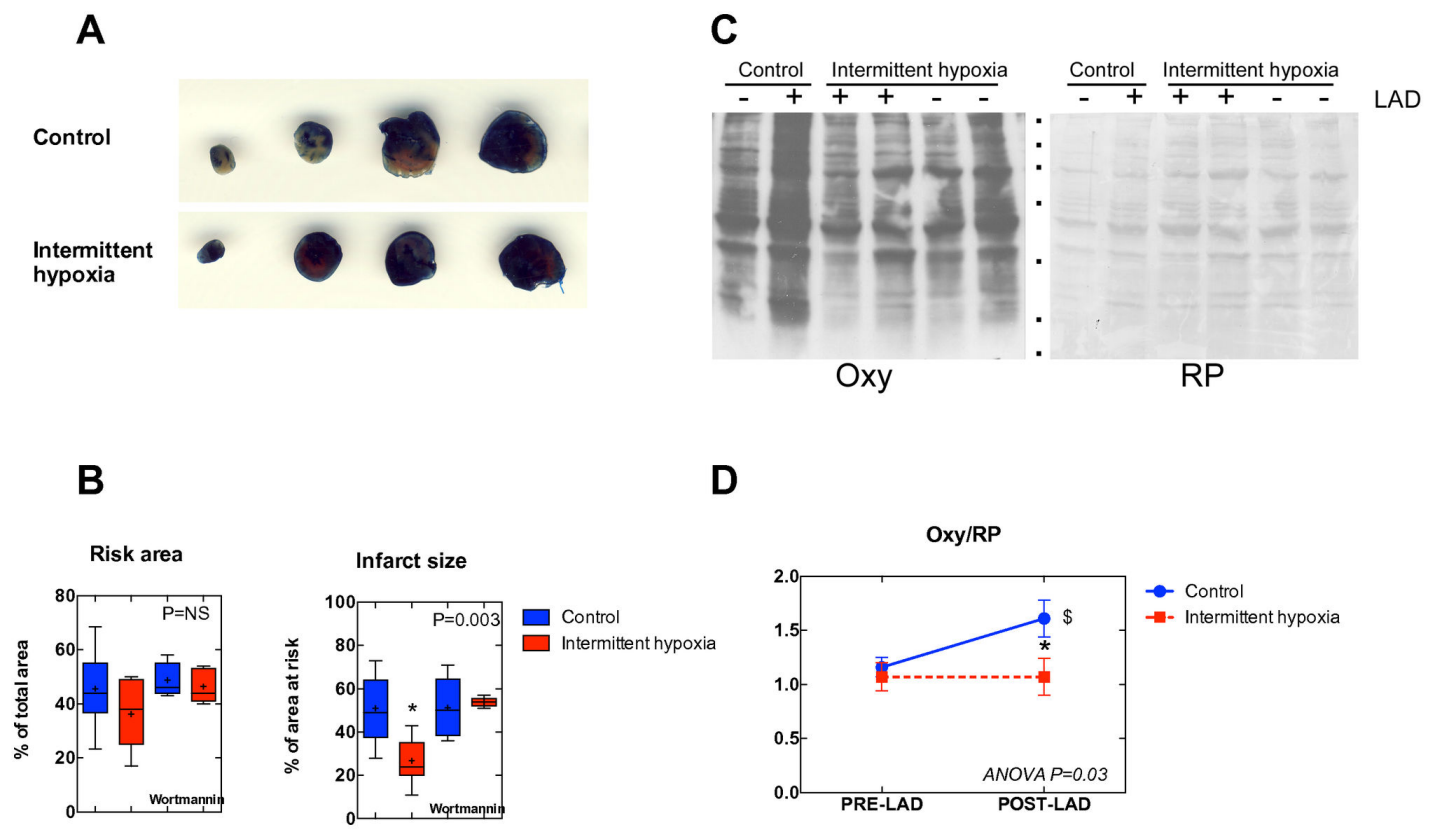
Cardioprotection. Panel A. Representative images taken in a control and a IH heart after staining myocardial tissues slices with triphenyltetrazolium chloride to mark the infarct and risk areas. Whereas the blue and white areas represent viable and necrotic tissues, respectively, the red+white area represents the area at risk. Panel B. Box-and-whisker plots from all the hearts subjected to LAD occlusion and reperfusion. The boxes represent the 2^nd^ and 3^rd^ quartiles of data, with the whisker delimiting the min-max range. The “+” represents the mean (n=10/7 and 5/5 without and with wortmannin, respectively). The insets report the ANOVA values. Panel C. Left: Representative Oxyblot (Oxy) analysis. Right: Loading of the same nitrocellulose sheet as visualized by Red Ponceau (RP) staining. Panel D. Normalized densitometry values (Oxy/RP), index of protein carbonylation. *, P<0.05 with respect to control; $, P<0.05 with respect to PRE-LAD (ANOVA one-way test, followed by the Bonferroni multiple comparison procedure, two tailed, the inset reports the value of the ANOVA P, n=6/6 and 8/5, pre-LAD and post-LAD, respectively).

Figure **6A** shows representative images taken in one control and one IH heart after staining myocardial tissues slices with triphenyltetrazolium chloride to mark the infarct and risk areas. Whereas the blue and white areas represent viable and necrotic tissues, respectively, the red+white area represents the area at risk. Figure **6B** shows the averaged risk and infarct areas of all the hearts subjected to LAD occlusion and reperfusion. Both IH and wortmannin did not change the risk zone, indicating that the extent of the LAD ligature was the same in all the groups. However, the infarct area was markedly decreased in IH, indicating the occurrence of IH-induced cardioprotection. The presence of wortmannin, on the other hand, blunted the protective effect of IH.

Cardioprotection was also evaluated by assessing the degree of protein carbonylation, an index of redox unbalance (Figure **6C**). Normalized densitometry values (Oxy/RP) increased markedly in myocardial tissue of control hearts compared to IH, reflecting the occurrence of oxidative stress in control but not in IH (P<0.01) (Figure **6D**).

## Discussion

The IH protocol described here enabled hearts to improve performance, induce neo-angiogenesis, increase the expression of HO-1 and decrease that of CHOP, indicative of attenuated apoptosis induced by endoplasmic reticulum stress. By contrast, the myocardial diastolic function and the expression levels of HSP70 and GRP94 were not affected by IH, indicative of lack of major adverse events originated by excess oxidative stress. Likewise, the hypoxia signaling pathway did not appear to be altered by IH, as indicated by lack of changes in HIF-1α. Nevertheless, Akt phosphorylation was markedly increased. Collectively, these effects led to two important hallmarks in cardiovascular protection. First, cardiac function was improved, in qualitative agreement with data obtained in healthy and transgenic (over-expression of tumor necrosis factor α, which induces heart failure) rodent models [23]. Second, cardioprotection was ameliorated as from reduced infarct size after LAD ligature and reduced formation of protein carbonyl groups in response to LAD. The administration of the PI3K inhibitor wortmannin suppressed Akt phosphorylation and some of the downstream effects, including eNOS and Nrf2, leaving HIF-1α unaffected. The inhibition led by wortmannin on Nrf2 expression, and hence the link between PI3K activity and the regulation of anti-oxidative proteins, has already been demonstrated [24]. Furthermore, wortmannin reversed the favorable effects led by IH in terms of infarct size reduction, indicating that the cardioprotection induced by IH appeared directly related to the IH-induced over-phosphorylation of Akt.

The measurement of the infarct area following LAD occlusion/reperfusion is related to the functional recovery of Langendorff-perfused hearts after I/R [10]. The LAD occlusion challenged ^≈^40% of the left ventricle in all groups, yet the infarct area at the end of the reperfusion was 50% of the risk area in control hearts, which reduced by half in IH hearts, as a result of effective cardioprotection. IH-induced cardioprotection was entirely reverted by previous administration of wortmannin. This outcome is in qualitative agreement with a study where IH was given by exposing rats to a single 4-h period of hypobaric hypoxia (barometric pressure=404 mmHg) each day for four weeks [25]. At odds with the present investigation, in that study rats did not experience any significant change in body mass nor improved baseline myocardial performance. Whereas those Authors focused into the post-conditioning-like effects led by IH addressing the role of mitochondria in generating sub-lethal doses of ROS early at the reperfusion after ischemia in Langendorff-perfused hearts [26], here we focus into the pre-conditioning effects of IH by addressing the role of a number of signaling pathways.

### IH and angiogenesis

Administering IH for 14 days markedly increased the capillary network. Electron microscopy demonstrated the formation of intercapillary pillars, which supports the hypothesis that neo-angiogenesis occurred mostly by intussusceptive growth, also known as splitting angiogenesis. This modality of capillary growth is characterized by capillary bed expansion through formation of finger-like inter-endothelial protrusions, which give rise to transcapillary tissue pillars forming a transluminar bridge, and eventually generate new vascular segments [27]. The intussusceptive angiogenesis, typical of developmental processes, generally coexists with vascular sprouting, the alternative modality of angiogenesis which predominates in cancer tissues [28]. Notably, intussusceptive capillary growth is fast (minutes-hours) as it does not primarily need cell proliferation. It is believed that an extended capillary network increases the blood supply and provides alternative shunt circulation on vessel obstruction, thus contributing to cardioprotection. In the described IH frame, neo-angiogenesis was accompanied by increased VEGF-R2 protein expression, whereas VEGF proteins increase was of borderline statistical significance, in analogy with observations gathered in aerobic exercise-trained rats [18]. VEGF-A, perhaps the most important VEGF isoform because of its critical role in the development of embryonic vasculature, binds to either one of the VEGF-R isoforms, but the VEGF-R2/flk-1 isoform is known to mediate the majority of cell responses to VEGF-A [29]. VEGF-A binding causes VEGF-R2 dimerization and activation through a pathway leading to PLC-γ activation, increase in cytoplasm Ca^++^, activation of protein kinase C and phosphorylation of at least two of the mitogen-activated protein kinases, e.g., extracellular signal-regulated kinases ERK1/2 and p38 [30], which then move to the nucleus where they participate to various transcriptional activities leading to cell proliferation and migration [31]. Of interest, an OSA-like IH paradigm increases VEGF immunoreactivity in the carotid body thereby enhancing carotid body chemosensory response to hypoxia [32].

VEGF and VEGF-R2 are known downstream effectors of the hypoxia-inducible factor (HIF)-1α, the master regulator of O_2_ homeostasis. Although it is likely that the over-expression of pro-angiogenesis factors depend on the myocardium response to hypoxia, we were unable to document significant changes in HIF-1α protein expression, perhaps due to the short hypoxia times followed by reoxygenation, which blunts HIF-1α protein over-expression [33]. We can’t exclude, however, the occurrence of fast peaks in HIF-1α protein levels in the correspondence of the hypoxia bouts. Indeed HIF-1α is likely to play a critical role, because acute IH (6 min 6% O_2_ + 6 min 21% O_2_, 5 times) gave protection that was lost in *Hif1α* ^*+/-*^ mice [34]. Albeit short, HIF-1α transactivation may nevertheless activate the transcription of a number of growth factors related to angiogenesis, including VEGF and its receptors. In mice constitutively expressing HIF-1α, neo-angiogenesis is pivotal to reduce infarct size and attenuate cardiac dysfunction through VEGF-A protein expression in infarct and peri-infarct areas [35]. As a matter of fact, however, the role of HIF-1α appeared marginal in this study, because treatment of wortmannin suppressed the cardioprotective role of IH leaving HIF-1α unaffected.

IH mice are exposed not only to short hypoxic events, but also to repetitive ROS bursts during the reoxygenation phase. Such bursts might up-regulate HIF-1α activity. For example, ROS activate PLC-γ signaling, thus increasing intracellular Ca^++^ and activating Ca^++^/calmodulin-dependent protein kinase (CaMK), which phosphorylates the HIF-1α co-activator p300 and promotes HIF-1α activation [36]. Moreover, CaMK and diacylglycerol induce the activity of PKC, which stimulates mTOR-dependent HIF-1α synthesis and inhibits its targeting to ubiquitin by inhibiting prolyl-hydroxylases [37]. Since the protection occurred after factors such as HIF-1α have presumably returned to baseline, protection is likely related more to morphological or persisting changes such as angiogenesis than to direct effects by HIF-1α. Although the role of neo-angiogenesis in promoting cardioprotection is unquestionable, there is however evidence that other signaling pathways may have a pivotal role in IH-induced cardioprotection.

### IH and stress response

The employed IH protocol is effective in increasing the expression of HO-1, a HIF-1α target [38]. However, IH does not affect the expression of the stress-proteins involved in the heat shock pathway or the ER-stress response, as demonstrated by lack of change in the amount of Hsp70 and Grp94. By contrast, IH reduces the expression of CHOP and increases that of Nrf2, a transcription factor that represents the primary defense against the cytotoxic effects of oxidative stress and increases the expression of several antioxidant enzymes. Furthermore, IH appeared to decrease CHOP in about half of cardiomyocytes. Although CHOP mRNA is not changed in hearts exposed to 5 h hypoxia (10 or 6.5% O_2_) [39], these findings might be explained with the evidence that CHOP expression and activation are selectively blunted after inhibition of prolyl-hydroxylases [40]. Prolyl-hydroxylases are inhibited by hypoxia and their inhibition increases the transcription factor Nrf2 [40], another positive regulator of HO-1 expression [38]. Since IH increases Nrf2 levels at variance with chronic hypoxia [41], it is likely that IH generates an inhibition of prolyl-hydroxylases that reveals sufficient to reduce nuclear localization of CHOP and increase the expression of HO-1. The oxidative stress associated with IH plays a role in the development of increased cardiac ischemic tolerance. The infarct size-limiting mechanism of IH seems to involve the PKC-delta-dependent pathway but apparently not the increased capacity of major antioxidant enzymes [42], and the transcriptional activation of HSP70. Increased Nrf2 levels seems to be crucial in the upregulation of HO-1, which has been reported to have the most antioxidant response elements on its promoter [43]. HO-1 activity also releases carbon monoxide, which increases the expression of cardioprotective and anti-apoptotic molecules [44].

A further consequence of decreased CHOP activation is the relatively low oxidative stress that accompanies LAD in IH compared to control hearts. Although the degree of protein carbonylation might simply reflect the size of the infarcted area, the possibility exists that the smaller size of the infarcts observed in IH hearts derives from decreased oxidative stress. Among its effects, CHOP positively regulates the expression of the potent ER oxidase Ero1alpha, leading to oxidative stress and apoptotic death [45]. It is therefore likely that the lower number of cardiomyocytes displaying nuclear CHOP localization in IH hearts represent a lesser source of oxidant production when ischemia disrupts ER function, since nuclear CHOP localization indicates the presence of a sustained ER-stress response [45].

### IH-induced signaling pathways

IH markedly activated Ser-473 Akt phosphorylation, which was blunted on administration of wortmannin, concomitantly with loss of IH-induced cardioprotection. This strongly suggests that the cardioprotection elicited by IH is mediated by the activation of the PI3K-Akt pathway. Hypoxia is not known as an Akt activator, but the reoxygenation after hypoxia enables cardiomyocytes to phosphorylate Akt [46] without affecting Akt expression [47]. As the reoxygenation of hypoxic tissue is associated to ROS bursts, ROS may act as triggers for Akt phosphorylation [48,49]. The link among P-Akt, hypoxia and cardioprotection is not universally acknowledged. In rats exposed to IH (12% O_2_, 8 h/day for 4 and 8 weeks) P-Akt decreases with increased apoptosis through both mitochondrial-dependent and Fas death receptor-dependent paths [50]. In cultured cardiomyocytes subjected to hypoxia and reoxygenation, induction of gene 33 mRNA and Gene 33 protein reduces Akt signaling [51]. Both these examples refer to IH situations that resemble the OSA-like rather than the cardioprotective paradigm, which reinforces the idea that cardioprotective IH is associated to enhanced PI3K-Akt signaling. When IH is given for 4 h (40 s at 10% O_2_ followed by 20 s at 21% O_2_), the resulting cardioprotection, assessed in Langendorff-perfused hearts, is largely due to the signaling downstream PKC, p38 MAPK and ERK1/2 without involving the PI3K signaling [52].

Several observations support a role for P-Akt to mediate cardioprotection, in addition to its known effect in hypertensive [53] and infarcted hearts [54]. A recognized factor that triggers hypoxic preconditioning [55], its blockade by the antagonist LY-294002 suppresses the cardioprotection induced by the reoxygenation of hypoxic hearts [47]. The cascade activated by Akt represents a common route in eliciting preconditioning, for example by activating the endothelial isoform of NO synthase [56] (see also Figure 4) and the NO/cGMP pathway, as observed in hypoxic brain tissue [57]. The beneficial effect of Akt activation on cardioprotection is evident in chronic hypobaric IH (8 h/day, 25-30 exposures) [58]. The reactivation of Akt has also been recognized as a critical determinant of survival in post-hypoxic cardiomyocytes in culture [59]. By contrast, in a minipig LAD model, the immunosuppressant tacrolimus, or FK-506 or fujimycin, a 23-membered macrolide lactone, was shown to limit infarct by suppressing the inflammatory response through down-regulation of the Akt signaling pathways [60], but the same substance also up-regulates HO-1, which is at odds with our observations. Interestingly, in an OSA model where IH is not protective, Akt Ser^473^ phosphorylation was found to be decreased [61], in agreement with present data. Akt activation may also provide protection via increased eNOS and augmented NO stores. In fact, the anti-hypertensive effect of IH in young spontaneously hypertensive rats is associated with prevention of endothelial dysfunction and with increased accumulation of NO stores in vascular walls due to augmented eNOS activity [62]. The ability of IH to increase eNOS is shared also in OSA-like models at the level of the carotid body [63]. Full understanding of the role of NO in IH, however, should include monitoring not only of the expression of Akt-activated eNOS, but also of transcriptionally-activated iNOS, which has been shown to play a key role in IH-induced delayed cardioprotection [64,65].

## Conclusion

IH treatment may represent an efficient and economic way to induce long-term preconditioning without use of drugs, and comparable to the protection induced by physical training. Here, we describe an IH protocol that enables hearts to improve performance, induce neo-angiogenesis and resist to I/R injury. We identified the PI3K-Akt signaling as a key path that mediates IH-induced cardioprotection. Such pre-conditioning would not necessarily reduce the incidence of myocardial I/R injury, but would likely affect the severity of myocardial damage.
